# The Medical Research Committee

**Published:** 1919-04-05

**Authors:** 


### THE MEDICAL RESEARCH COMMITTEE.

### Fourth Annual Report.

It is but a short time ago that arrangements were urged for the continued freedom of the Committee under the coming Ministry of Health, and their recent report comes in convincing justification of this requirement. In previous years the annual publication appeared at a time when the conditions of active warfare found the country in a state so unsettled that, although the stimulus to inquiry was present on every hand, fullest co-ordination of results could not be achieved, but on this occasion circumstance has allowed for a very thorough review of the immediate situation. The report provides a first comprehensive outline of all that the exigencies of war have meant to the advancement of medical science. One sees an invaluable sketch of the foundations upon which the new and much hoped for era of investigation and preventive medicine may be based.

It is pointed out that during these years of stress, when the power of every science has been brought to bear on mankind for either his destruction or salvation, there have, in the latter cause, been two significant influences. Firstly, in all previous European wars, deaths from infecting organisms have greatly exceeded those from enemy weapons, but in this, the greatest conflict of all time, the relation has been entirely reversed by bacteriological study. It is possible in some measure to estimate, but in imagination hard to realise, the enormous amount of human suffering that has been saved. Secondly, the physiologist has successfully dealt with innumerable problems arising from violence and strain to millions of human bodies. In fatigue and exposure, nervous tension, physical injury, the incredible stress of high altitude aviation and air fighting, this science has been brought into the closest touch with medical work, and, apart from its immediate achievements, we see the first steps towards the urgent and necessary reunion between the laboratory and the clinician.

The Committee have done everything possible to encourage and develop "team work," not in Great Britain alone, but also in conjunction with France, America, and Italy. Even at this early stage of peace preparation their success has been admirable. During the war opportunity has from time to time been taken to provide our Government departments with translations and abstracts from the medical statistics and experiences of other countries. Finally, arrangements were made that their publication, the *Medical Supplement*, should deal chiefly with enemy countries and act as the complement of the *Medical Bulletin*, issued by the American Red Cross Research Committee and containing a review of Allied work of particular military interest. This scheme has been extended and elaborated until the two volumes (N.B.—the American is now called *War Medicine*) should form a complete and up-to-date *précis* of medical research work the world over.

It is impossible in a few words to mention more than one or two of the outstanding sectional reports. Almost without exception they indicate an opening up of new fields and a rate of progress of which this country may be justly proud. In the continued work upon the assymmetrical forms of T.N.T., the properties of the substances in forming condensation products with amino-acids have been used to elucidate the structure of carnosine, a polypeptide found native in the human body. This substance, a complicated natural product of metabolism, is thus for the first time synthetised in the laboratory, and provides an excellent example of the way in which inquiry undertaken for a purely practical purpose—T.N.T. poisoning—may lead to unexpected results in quite other fields of science.

Among the schemes of research framed before the war, it is seen that most have been continued and advanced. Tuberculosis, the pathology of infection, its treatment by tuberculins and new specific methods, and the occupational incidence of the disease are all under consideration, while the excellent work on dietetic disease, referred to in connection with rickets on another page of this journal, is progressing admirably. As a result of a study of anærobic wound infection and gas gangrene, an efficient anti-serum has been developed, the decision being made immediately to introduce its administration on the same lines as adopted against tetanus in the early phases of the war. The efforts of several laboratories are now devoted to the production of large quantities of serum with high protective power against *B. welchii*, *vibrion septique*, and *œdematiens*.

A further bacteriological advance and one suggestive of the greatest possibilities, lies in the expression in precise physico-chemical terms of the nutritive conditions which determine the activity of growth and the toxin production of special types of bacteria. Laboratory technique becomes more and more complicated, but differentiation of germs and the experimental production of immunity, serums, and vaccines are immensely improved.

Practically every disease of peace and war is in some way under active consideration by these workers, and most interesting announcements and reviews should be heard at the forthcoming clinical meeting of the B.M.A., where many of the Committee's investigators will speak or lecture. One trusts that the urgency of war will not for many years again stagger the civilised world, and that the outcome of this mass of strenuous but painstaking research may now be turned to the use of the civil population. Their needs are too little realised, and in the words of the report "long familiarity tends to deaden the sense of the community to the immense toll of life and health levied by removable causes in peace time upon our population." The foundation of an enormous amount of preventive work has been laid, and before commencing its building we can afford not one moment of unnecessary delay.

